# Into the pancreas: an underwater world filled with an intraductal papillary mucinous neoplasm seen via peroral pancreatoscopy

**DOI:** 10.1055/a-2339-2121

**Published:** 2024-06-26

**Authors:** Kazuki Hirano, Kosuke Maehara, Daisuke Hattori, Yoshiki Sato, Tetsuo Tamura, Rikako Koyama, Tsunao Imamura

**Affiliations:** 1 13600 Department of Gastroenterology, Toranomon Hospital, Minato-ku, Japan; 2 545161 Okinaka Memorial Institute for Medical Research, Minato-ku, Japan

Although peroral pancreatoscopy (POPS) has been developed for detailed visualization of intraductal lesions, the images obtained are often inadequate because of the difficulty of insertion, the proximity of the pancreatoscope to the lesion, or visualization challenges due to mucus. Performance can be improved when it is combined with intraductal ultrasonography (IDUS) 1 , narrow-band imaging (NBI) 2 , and probe-based confocal laser endomicroscopy (pCLE) 3 ; however, POPS alone has not achieved high quality intraductal imaging and is still under development.

Here, we report for the first time the use of an ultrathin endoscope with a transparent hood at the tip (Nichendo; Fujifilm Co., Tokyo, Japan) in POPS. This technique has been used in endoscopic submucosal dissection 4 ; however, its use in the pancreaticobiliary region has not been reported.

A man in his 80s underwent follow-up magnetic resonance cholangiopancreatography (MRCP) for a branch duct intraductal papillary mucinous neoplasm (BD-IPMN); a fistula between the pancreatic duct branch and the stomach was suspected, with gastric perforation of the BD-IPMN ( Fig. 1 ). To obtain a definitive diagnosis, we initially attempted visualization with a conventional endoscope (GIF-H290Z; Olympus, Tokyo, Japan); however, insertion was difficult ( Fig. 2 ). Therefore, an ultrathin endoscope (GIF-XP260N; Olympus) with a transparent hood was used ( Fig. 3 , Video 1 ).

**Fig. 1:**
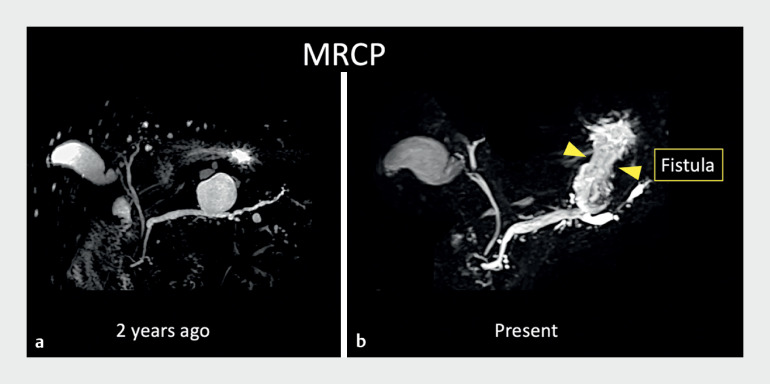
Images from magnetic resonance cholangiopancreatography of an elderly man with branch duct intraductal papillary mucinous neoplasm (BD-IPMN) performed: **a** 2 years previously; **b** during this presentation, with a fistula between the pancreatic duct branch and the stomach suspected.

**Fig. 2:**
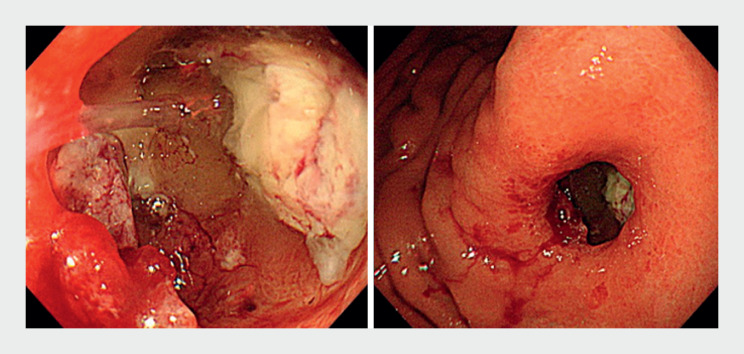
Images of the views obtained with a conventional endoscope (GIF-H290Z; Olympus, Tokyo, Japan).

**Fig. 3:**
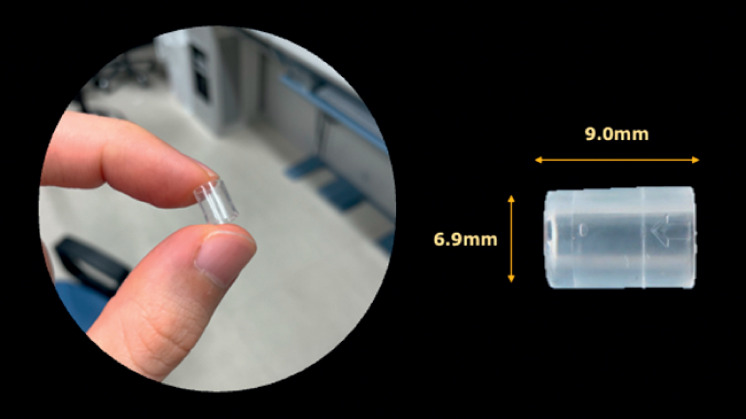
Photograph of the transparent hood (Nichendo; Fujifilm Co., Tokyo, Japan), which has an outer diameter of 6.9 mm and a length of 9 mm and creates a clear and direct perspective.

An ultrathin endoscope with a transparent hood attached to the tip was used to perform peroral pancreatoscopy via a fistulous tract, yielding high quality intraductal pancreatic images, improved insertion, and a stable field of view. Source for the underwater photographs: ACworks Co. Ltd. Maho Ishikawa.Video 1

This method enabled visualization of the pancreatic duct and its branches. White-light endoscopy (WLE) and NBI allowed visualization of the mucosa with high quality images and suggested a relationship between the mucosal appearance and atypical tissue ( Fig. 4 ). Characteristic findings including a “bleached coral-like appearance”, indicating rough mucosa, “jellyfish-like appearance” at the entrance of the pancreatic branch duct, and “anemone-like appearance” near the gastrostomy were seen ( Fig. 5 ).

**Fig. 4:**
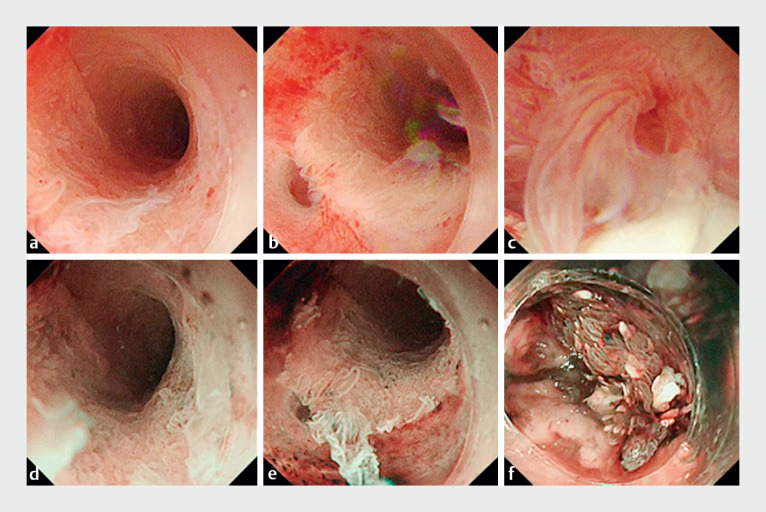
Characteristic mucosal appearances seen on: **a–c** white-light endoscopy; **d–f** narrow-band imaging for areas with: **a, d** low grade mucosal changes; **b, e** high grade changes; **c, f** invasive carcinoma.

**Fig. 5:**
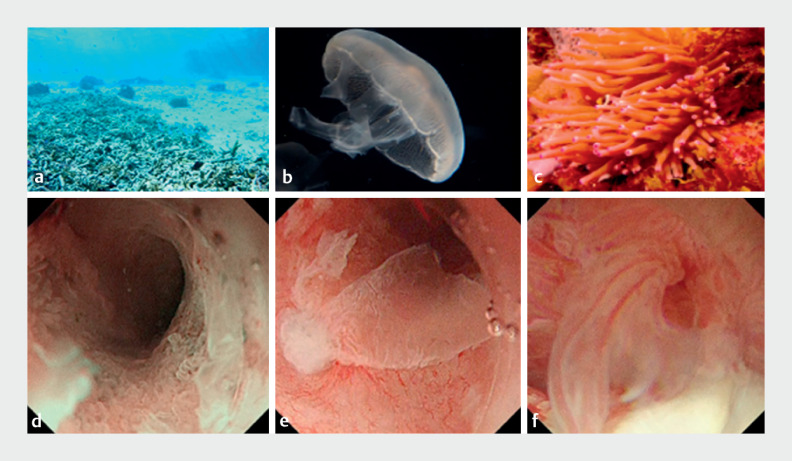
Mucosal images on peroral pancreatoscopy, along with their underwater likenesses, showing: **a, d** the bleached coral-like appearance of low, rough mucosa inside the pancreatic duct; **b, e** the jellyfish-like appearance of fragile mucosa floating at the entrance of the pancreatic duct; **c, f** the sea anemone-like appearance of a tall, highly atypical mucosa near the gastrostomy. Source for Figure 5 **a** and **c** : ACworks Co. Ltd. Maho Ishikawa.

This is the first report of an ultrathin endoscope with a transparent hood being used for POPS, providing high quality intraductal pancreatic images, improved insertion, and a stable field of view.

Endoscopy_UCTN_Code_CCL_1AZ_2AB

## Notes

Endoscopy E-Videos https://eref.thieme.de/e-videos
*E-Videos* is an open access online section of the journal *Endoscopy* , reporting on interesting cases and new techniques in gastroenterological endoscopy. All papers include a high-quality video and are published with a Creative Commons CC-BY license. Endoscopy E-Videos qualify for HINARI discounts and waivers and eligibility is automatically checked during the submission process. We grant 100% waivers to articles whose corresponding authors are based in Group A countries and 50% waivers to those who are based in Group B countries as classified by Research4Life (see: https://www.research4life.org/access/eligibility/ ). This section has its own submission website at https://mc.manuscriptcentral.com/e-videos .
